# Pharmacoeconomic evaluation of venlafaxine compared with citalopram in generalized anxiety disorder

**DOI:** 10.3892/etm.2012.869

**Published:** 2012-12-20

**Authors:** JINGJING ZHANG, HONGBING XU, ZHIQING CHEN

**Affiliations:** Department of Psychological Medicine, Shanghai First People’s Hospital, Shanghai 200080, P.R. China

**Keywords:** generalized anxiety disorder, venlafaxine, citalopram, cost-effectiveness analysis

## Abstract

Pharmacoeconomic evaluation aims to investigate the selection and use of drugs to make patient medication efficient, safe and economical. In this study, a pharmacoeconomic evaluation was performed to assess two treatments for generalized anxiety disorder (GAD). A total of 100 outpatients with GAD were enrolled. The patients were divided into the following two groups according to treatment program: the venlafaxine group (n=50) and the citalopram group (n=50). The patients in the venlafaxine group received 75 mg of orally administered venlafaxine and 50 mg of sulpiride once a day for the first seven days and thereafter only 150 mg of venlafaxine once a day. The patients in the citalopram group received 10 mg of citalopram and 50 mg of sulpiride orally once a day for the first seven days and thereafter only 20 mg once a day. The treatment period for the two groups was three months. Follow-up was conducted at the end of weeks 2, 4 and 12 to evaluate drug efficacy, quality of life and drug side-effects. Moreover, the two groups were scored according to cost-effectiveness analysis. Using the SF-36 Scale, the quality of life score of the patients in the venlafaxine group was observed to be significantly higher compared with that of the patients in the citalopram group at the end of weeks 4 and 12 (P<0.05). The reduction rates of the Hamilton Anxiety (HAMA) scale show that the efficacy of venlafaxine was significantly better than that of citalopram by the end of week 12. The findings of this study suggest that venlafaxine is more cost-effective than citalopram in the treatment of outpatients with GAD.

## Introduction

Anxiety disorder is one of the most common mental disorders handled in general hospitals. Anxiety disorder was recently reported to have prevalence of 5.6% (5.0–6.3%) in several regions of China (1). Generalized anxiety disorder (GAD) is a disabling disease often diagnosed with other mental disorders, and thus causes a heavy burden on patients (2). In current clinical practice, the major therapeutic drugs for GAD include selective serotonin reuptake inhibitors (SSRI) and serotonin and norepinephrine reuptake inhibitors (SNRI). GAD requires long-term treatment of at least 12 months in the first instance (3–5). Given the prolonged duration of the treatment, clinical practitioners should consider cost-effectiveness when selecting therapeutic plans. Citalopram and venlafaxine, which are reasonably priced drugs covered by Medicare, are two well-accepted drugs for GAD treatment. Doctors and patients alike are willing to use citalopram and venlafaxine, making these drugs comparable for study. In the present study, a quality of life scale was employed to thoroughly evaluate and examine the efficacy of the two treatments (6).

## Patients and methods

### Subjects

Between January 2010 and June 2011, outpatients at the Shanghai First People’s Hospital (Shanghai, China) who had been diagnosed with GAD according to the criteria defined in the fourth edition of the Diagnostic and Statistical Manual of Mental Disorders (DSM-IV) (7) were approached by the research staff of the study. The inclusion criteria were outpatients who were aged between 18 and 65 years old and who had Hamilton Anxiety (HAMA) scale scores >14. Patients diagnosed with other mental disorders and serious heart, liver and kidney damage were excluded from the present study. Overall, 100 patients, including 42 males and 58 females, aged 44.26±11.34 years old, were enrolled. The researchers assigned the 100 subjects into two treatment groups, namely, the citalopram group and the venlafaxine group. The citalopram group comprised 50 patients, including 20 males and 30 females, aged 43.48±12.3 years old, with Symptom Checklist-90 (SCL-90) scores of 185.90±32.32, HAMA scores of 19.12±2.21 and Self-Rating Anxiety Scale (SAS) scores of 39.00±8.36. The venlafaxine group comprised 50 patients, including 21 males and 29 females, aged 45.04±10.28 years old, with SCL-90 scores of 193.06±43.13, HAMA scores of 19.02±2.49 and SAS scores of 40.72±7.43. No significant difference was observed between the two groups in the previously mentioned statistics (P>0.05). Family members or the patients themselves signed an informed consent form prior to participation in the present study.

### Method of administration

For the citalopram group, the treatment plan was 10 mg citalopram (trade name, Cipramil; tablets, 20 mg; Xi’an Janssen Pharmaceutical Co, Ltd., Xi’an, China) and 50 mg sulpiride once a day for the first seven days and thereafter only 20 mg citalopram once a day. The treatment period was three months.

For the venlafaxine group, the treatment was 75 mg venlafaxine (trade name, Efexor; capsules, 150 mg; Pfizer Pharmaceutical Co, Ltd., New York, NY, USA) and 50 mg sulpiride once a day for the first seven days and thereafter only 150 mg venlafaxine once a day. The treatment period was three months.

### Measures

At the end of 2, 4 and 12 weeks of treatment, two senior psychological physicians from the Shanghai First People’s Hospital evaluated the efficacy of the two treatments and recorded the adverse reactions with reference to the HAMA, SAS, health status questionnaire (SF-36) and side-effects of antidepressants scale (SERS). In relation to the endpoint, HAMA remission (total HAMA score ≤7) was considered to be a clinical cure and an improvement in the HAMA response of ≥50% from the baseline was considered to be effective (8).

The self-made cost estimates scale included registration fees, inspection fees, drug fees and other direct medical costs, as well as lost wages, adverse reaction treatment and alternative treatment costs (9). The following formula was used: Loss of working time fee = the average income in Shanghai in 2009/365 x days of working time lost. During the course of treatment, it was necessary to change the treatment programs of several cases due to serious adverse reactions. Seroxat (20 mg po qd) was used as an alternative treatment regimen.

### Statistical analysis

The SPSS 10.0 statistical software (SPSS, Inc., Chicago, IL, USA) was used to obtain data statistics. The t-test and the the Chi-square test for categorical variables were used for comparisons between the two groups. P≤0.05 was considered to indicate a statistically significant difference.

## Results

### Comparison of the HAMA scores of the two groups before and after treatment

The HAMA score reduction rates of the two groups gradually increased during the treatment. In addition, the HAMA scores before and after treatment were significantly different (P<0.01) within each group. No significant difference (P>0.05) was observed in the HAMA scores of the two groups prior to treatment. However, a significant difference (P<0.01) in the HAMA reduction rates of the two groups was observed following two weeks of treatment. Moreover, the anti-anxiety effect of venlafaxine was observed to be greater than that of citalopram. After 4 and 12 weeks of treatment, significant differences (P<0.01) in the HAMA reduction rates of the two groups remained evident. Moreover, the reduction rate of the venlafaxine group was observed to be greater than that of the citalopram group at both time points (Table I).

### Comparison of the SF-36 scores of the two groups before and after treatment

No significant difference was observed in the quality of life scores of the patients in the two treatment groups prior to treatment (P>0.05). The SF-36 scores of the two groups increased following two weeks of treatment. Moreover, the SF-36 scores (P<0.01) were significantly different before and after treatment within each group. Significant differences (P<0.01) in the SF-36 scores of the two groups were evident following 4 and 12 weeks of treatment. Furthermore, the SF-36 scores of the venlafaxine group were observed to be greater than those of the citalopram group (Table II). The quality of life of the patients in the venlafaxine group was superior to that of the patients in the citalopram group.

### SERS ratings of the two groups before and after treatment

After 2, 4 and 12 weeks of treatment, the SERS ratings of the venlafaxine group were 1.58±1.70, 1.02±1.62 and 0.88±1.66, respectively and those of the citalopram group were 1.52±1.42, 1.32±1.38 and 0.76±1.15, respectively. Based on these results, the two groups were not significantly different in terms of SERS ratings (P>0.05; Table III).

In the venlafaxine group, 28 patients exhibited adverse reactions, including gastrointestinal discomfort, nausea, high blood pressure, dizziness, sleepiness and physical fatigue. In the citalopram group, 26 patients exhibited adverse reactions, including dizziness, body fatigue, drowsiness, gastrointestinal discomfort and nausea.

### Comparison of the clinical efficacies in the two groups

Reduction rates of >50% in HAMA rating scores serve as clinically valid values for the evaluation of efficacy. After 2, 4 and 12 weeks of treatment, the efficacies of the venlafaxine treatment were 38, 82 and 94%, respectively, whereas those of the citalopram treatment were 20, 56 and 70%, respectively. Using Chi-square test, no significant differences (P<0.05) in the clinical efficacies in the two groups were observed following two weeks of treatment. However, significant differences (P<0.01) in the clinical efficacies in the two groups were observed after 4 and 12 weeks of treatment (Table IV).

### Comparison of the costs of the two groups

Starting from the initial treatment, the treatment costs of the venlafaxine group were greater those of the citalopram group at each observation time point. The average total cost of each treatment case in the venlafaxine group was 1644.28±106.90 after 12 weeks of treatment. However, the average total treatment cost of each treatment case in the citalopram group was 1332.45±139.05 RMB, (t-test, P<0.01; Table V).

### Cost-effect analysis

Cost-effect analysis was performed to balance the cost and the effect and find an optimal point between them by linking the ratios of the cost and effect together. This ratio was represented by the cost of the unit effect. When we compare different treatment programs, some treatment programs with high efficacy are expensive, and as a result patient expenditure is increasing together with the treatment efficacy. This is why it is important to consider the cost of each treatment unit. Moreover, the cost per additional unit of efficacy, that is, the additional cost-effectiveness ratio (ΔC/ΔE), also requires consideration (Table VI).

## Discussion

GAD is a common clinical, chronic and recurring psychological disorder handled in general hospitals. With regard to GAD patients, clinicians endeavour to select treatments with good efficacy, rapid action onset and few and mild adverse reactions. Moreover, clinicians also require cheap drugs to improve treatment compliance and consequently increase the long-term treatment efficacy. Citalopram acts as an SSRI by increasing the concentration of serotonin (5-HT) in the synaptic cleft, which has anti-anxiety effects. Venlafaxine acts as an SNRI by increasing the levels of 5-HT and norepinephrine (NE) in the synaptic cleft to achieve therapeutic anti-anxiety effects. The results of the present study show that citalopram and venlafaxine are effective in GAD patients. Following 12 weeks of treatment, 94% of the patients in the venlafaxine group and 70% from the citalopram group were cured. The HAMA scores of the two groups were significantly different during the second, fourth and twelfth weeks of treatment. The SF-36 scores obtained during the fourth week were significantly different from those during the twelfth week. The patients in the venlafaxine group exhibited a superior improvement in their quality of life. The SERS scores of the three observation time points showed no significant differences between the two groups and the adverse reactions in the two groups were fairly mild. The venlafaxine treatment of GAD patients exhibited rapid efficacy and significantly improved quality of life. These characteristics may be attributed to the fact that venlafaxine acts not only on the 5-HT transporter but also on the NE transporter. In addition, GAD patients have slow clonidine reactions. After using yohimbine, NE metabolite 3-methoxy-4-hydroxyphenylglycol (MHPG) reaction in GAD patients is lower than in normal individuals. This indicated that the α2 adrenergic sensitivity of GAD patients is reduced (10).

The expenses of the treatments included the registration fees, inspection fees, psychological consulting fees and drug costs, as well as other direct and indirect medical costs. Indirect medical costs include loss of wages, adverse reaction treatment costs and the costs of replacement therapy following treatment failure. Understanding these costs is likely to aid the management of treatment failure and its impact on overall health care costs. Based on the results, the cost of treatment in the venlafaxine group was higher than that in the citalopram group. Moreover, the average cost of each treatment case in the twelfth week of treatment was ∼300% more than that at the start of treatment. Pharmacoeconomic analysis observed that venlafaxine treatment costs 17.49 RMB per unit, whereas citalopram treatment costs 38.06 RMB per unit. Each treatment unit of citalopram and venlafaxine costs >20.57 RMB. Therefore, clinicians choose venlafaxine to treat GAD patients with greater symptoms of severe anxiety since venlafaxine is more efficient than citalopram. In addition, venlafaxine improves the quality of life of the patients by a greater amount than citalopram (11). With regard to the cost to the patient, citalopram is also a good option due to its curative efficiency of ∼70%.

The present study was open-label and the observation period lasted for only 12 weeks. Therefore, continuous follow-ups to investigate the long-term efficacy and adverse reactions are necessary.

## Figures and Tables

**Table I.** HAMA scale scores of the two groups before and after treatment.

Time	Venlafaxine group (n=50)	Citalopram group (n=50)
Pretreatment	19.02±2.49	19.12±2.21^a^
Week 2	11.00±4.13^c^	14.04±4.11^b,^^c^
Score reduction	8.02±4.71	5.08±4.18^b^
Reduction rate	42.17	26.57^b^
Week 4	6.30±3.07^c^	8.62±3.57^b,^^c^
Score reduction	12.88±4.13	10.02±3.84^b^
Reduction rate	67.72	52.41^b^
Week 12	3.84±3.56^c^	6.64±3.57^b,^^c^
Score reduction	15.34±5.53	12.34±4.14^b^
Reduction rate	80.65	64.54^b^

a P>0.05,

b P<0.01 vs. the HAMA score of the venlafaxine group;

c P<0.01 vs. the pretreatment HAMA score. P-values were determined using a t-test. HAMA, Hamilton anxiety. HAMA scores are the means ± SD. Reduction rates are percentages (%).

**Table II.** SF-36 scores of the two groups before and after treatment.

Time	Venlafaxine group (n=50)	Citalopram group (n=50)
Pretreatment	360.52±135.71	409.76±136.31^a^
Week 2	577.05±131.50^c^	557.37±140.35^a,c^
Week 4	754.75±140.56^c^	626.92±141.34^b,^^c^
Week 4	802.41±122.56^c^	703.28±131.36b^c^

Comparison of the SF-36 scores of the two groups using a t-test,

a P>0.05,

b P<0.01; Comparison of the SF-36 scores of the two groups before and after treatment using a t-test,

c P<0.01. Scores are the means ± SD.

**Table III.** SERS ratings of the two groups after treatment.

Time	Venlafaxine group (n=50)	Citalopram group (n=50)
Week 2	1.58±1.70	1.52±1.56^a^
Week 4	1.02±1.62	1.32±1.38^a^
Week 12	0.88±1.66	0.76±1.15^a^

Comparison of the SERS ratings using a t-test,

a P>0.05. SERS, side effects of antidepressants scale. Ratios are the means ± SD.

**Table IV.** Comparison of the clinical efficacies of the two treatment programs.

	Venlafaxine group (n=50)		Citalopram group (n=50)	
Time	Clinical cure (%)	Effective (%)	Clinical cure (%)	Effective (%)
Week 2	4 (8)	19 (38)	1 (2)	10 (20)^a^
Week 4	19 (38)	41 (82)	8 (16)	28 (56)^b^
Week 12	33 (66)	47 (94)	16 (32)	35 (70)^b^

Comparison of the clinical efficacies of two groups using the Chi-square test:

a P<0.05,

b P<0.01.

**Table V.** Comparison of the treatment costs of the two groups.

Time	Venlafaxine group	Citalopram group
Week 0	398.66±24.84	344.28±34.60^b^
Week 2	258.36±55.51	189.65±47.57^b^
Week 4	252.60±12.88	212.30±94.34^b^
Week 12	734.66±119.98	586.22±106.69^b^
Total	1644.28±106.9	1332.45±139.05^b^

Comparison of the treatments costs of the two groups using a t-test:

a P<0.05,

b P<0.01.

**Table VI.** Cost-effect analysis of the two groups.

Group	Total cost	Effect (%)	C/E	ΔC/ΔE
Venlafaxine	1644.28	94	17.49	12.99
Citalopram	1332.45	70	38.06	

C/E, cost/effect;ΔC/ΔE, increased cost-effect ratio, that is increase of expenditure per increased effect unit. Costs were counted in RMB.

## References

[1] Phillips MR, Zhang J, Shi Q (2009). Prevalence, treatment, and associated disability of mental disorders in four provinces in China during 2001–05: an epidemiological survey. Lancet.

[2] Lieb R, Becker E, Altamura C (2005). The epidemiology of generalized anxiety disorder in Europe. Eur Neuropsychopharmacol.

[3] Reinhold JA, Mandos LA, Rickels K, Lohoff FW (2011). Phamacological treatment of generalized anxiety disorder. Expert Opin Pharmacother.

[4] Lenze EJ, Mulsant BH, Shear MK (2005). Efficacy and tolerability of citalopram in the treatment of late-life anxiety disorders: results from an 8-week randomized, placebo-controlled trial. Am J Psychiatry.

[5] Baldwin DS, Polkinghorn C (2005). Evidence-based pharmacotherapy of Generalized Anxiety Disorder. Int J Neuropsychopharmacol.

[6] Greener MJ, Guest JF (2005). Do antidepressants reduce the burden imposed by depression on employers?. CNS Drugs.

[7] American Psychiatric Association (1994). The Diagnostic and Statistical Manual of Mental Disorders.

[8] Pollack MH, Kornstein SG, Spann ME, Crits-Christoph P, Raskin J, Russell JM (2008). Early improvement during duloxetine treatment of generalized anxiety disorder predicts response and remission at endpoint. J Psychiatr Res.

[9] Eddama O, Coast J (2008). A systematic review of the use of economic evaluation in local decision-making. Health Policy.

[10] Krystal JH, Deutsch DN, Charney DS (1996). The biological basis of panic disorder. J Clin Psychiatry.

[11] Baca Baldomero E, Rubio-Terrés C (2006). Cost-effectiveness of venlafaxine for the treatment of depression and anxiety. Bibliographic review. Actas Esp Psiquiatr.

